# Are patient volume and care level in teaching hospitals variables affecting clinical outcomes in adult intensive care units?

**DOI:** 10.31744/einstein_journal/2023AO0406

**Published:** 2023-09-15

**Authors:** Rosane Milet Passos Teixeira, Jussiely Cunha Oliveira, Marcos Alécio Bispo de Andrade, Fernanda Gomes de Magalhães Soares Pinheiro, Rita de Cássia Almeida Vieira, Eduesley Santana-Santos

**Affiliations:** 1 Universidade Tiradentes Aracaju SE Brazil Universidade Tiradentes, Aracaju, SE, Brazil.; 2 Universidade Federal de Sergipe São Cristovão SE Brazil Universidade Federal de Sergipe, São Cristovão, SE, Brazil.

**Keywords:** Critical care, Intensive care units, Treatment outcome, Patients, Hospitals, teaching

## Abstract

**Objective:**

To compare the clinical outcomes of patients admitted to the intensive care unit of teaching (HI) and nonteaching (without an academic affiliation; H2) hospitals.

**Methods:**

In this prospective cohort study, adult patients hospitalized between August 2018 and July 2019, with a minimum length of stay of 24 hours in the intensive care unit, were included. Patients with no essential information in their medical records to evaluate the study outcomes were excluded.

**Results:**

Overall, 219 patients participated in this study. The clinical and demographic characteristics of patients in H1 and H2 were similar. The most prevalent clinical outcomes were death, need for dialysis, pressure injury, length of hospital stay, mechanical ventilation >48 hours, and infection, all of which were more prevalent in the teaching hospital.

**Conclusion:**

Worse outcomes were more prevalent in the teaching hospital. There was no difference between the institutions concerning the survival rate of patients as a function of length of hospital stay; however, a difference was observed in intensive care unit admissions.

## In Brief

Teixeira et al. showed that patients admitted to the intensive care unit of a teaching hospital in a non-metropolitan region needed more support, had worse prognostic indices, and had a higher nursing workload in the first 24 hours of admission. In addition, worse outcomes, including mortality, need for dialysis, pressure injury, infection, prolonged mechanical ventilation, and prolonged hospital stay, were observed in the teaching hospital.

## Highlights

Worse outcomes were more prevalent in the teaching hospital.

Understanding the importance of teaching hospitals to implement well-established care protocols is critical.

## Graphical abstract

**Figure:**
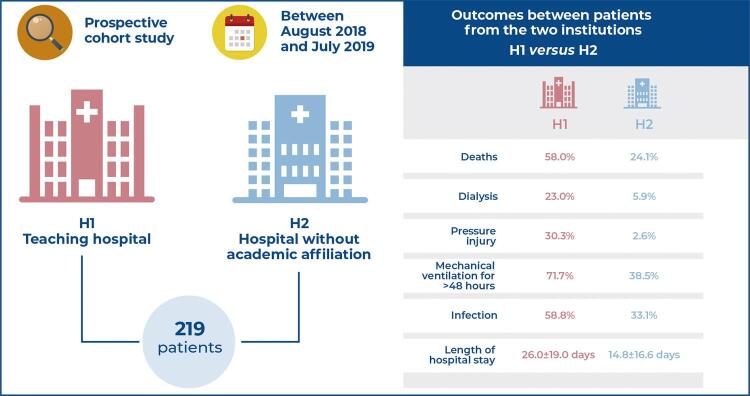

## INTRODUCTION

Some studies have reported the association between receiving hospital characteristics and the number of patients assisted with clinical outcomes.^(1,2)^ Some authors have also demonstrated this relationship in scenarios such as coronary care units and care units for patients with diabetes.^(3,4)^ Reportedly, hospitals with high admission volumes have better outcomes for surgical procedures; however, studies on the impact of volume on patient outcomes in the intensive care units (ICU) are limited.^(5,6)^

Some studies have attempted to associate better patient outcomes with different hospital settings, such as teaching and nonteaching hospitals. In a teaching environment, procedures are performed by students who may lack adequate expertise; therefore, potential unnecessary risks to patients exist. On the contrary, some studies have indicated superior results in teaching environments.^(7-9)^

Teaching hospitals are responsible for training health professionals and are the centers of clinical excellence in the era of evidence-based practice.^(10)^ Studies on the differences in quality of care between teaching and nonteaching hospitals have shown conflicting results, with some proposing that teaching hospitals are associated with increased costs, increased number of readmissions, and 30-day mortality. However, these findings may be associated with the greater complexity of the procedures performed in these settings and not with the quality of care.^(11,12)^

## OBJECTIVE

To determine whether patient volume and care level in teaching hospitals are variables affecting clinical outcomes in adult intensive care units.

## METHODS

This prospective cohort study was conducted in two ICUs in a non-metropolitan region of the state of Sergipe, northeastern Brazil. The *Hospital Universitário de Lagarto* (H1), located in the central-southern region of Sergipe, is a teaching hospital, and it attends to the spontaneous demands of the patients in that region. The *Hospital Regional de Itabaiana Dr. Pedro Garcia Moreno* (H2), located in the Agreste region, is a regional hospital with no academic affiliation. In addition to in-patient care, it provides urgent and emergency care 24 hours a day and outpatient services to the resident and transitory population and to those agreed with other municipalities. Both were medium-sized hospitals and were the only hospitals with an adult ICU outside the capital at the time of data collection.

Patients aged ≥18 years with a minimum length of stay of 24 hours in the two ICUs between August 2018 and July 2019 were included in the study. Those with no essential information in their medical records to assess the research outcomes were excluded.

For data collection, a specific instrument was developed, which included information on clinical and demographic characteristics and Simplified Acute Physiology Score 3 (SAPS 3) scores to assess severity. SAPS 3 admission score using patient characteristics, indications for ICU admission, and physiological derangement at ICU admission predicts hospital mortality. The Sequential Organ Failure Assessment (SOFA) is a scoring system that assesses the performance of several organ systems in the body (neurologic, blood, liver, kidney, and blood pressure/hemodynamics) and assigns a score based on the data obtained in each category.^(13)^ The Charlson Comorbidity Index (CCI) was used to assess the risk of death within 10 years and to categorize the comorbidities of patients based on the International Classification of Diseases (ICD) diagnosis codes found in the administrative data, such as hospital abstracts. The Nursing Activities Score (NAS) was used to assess the nursing workload at the patient level, considering the average time consumption for therapeutic and nursing activities, such as hygiene, mobilization, administrative activities, psychological support for patients and families, and patient care.^(12-17)^ The clinical and demographic characterization of the patients was performed by analyzing the following variables: age, sex, origin, presence of comorbidities according to ICD-10, laboratory test results, support for admission to the ICU, length of ICU stay, and mortality. The prognosis was estimated using SAPS 3 Scores calculated at admission and on day 7 or at discharge/death, whichever occurred first. The SOFA Score was calculated from the first 24 hours of ICU admission. Workload, measured by NAS, was evaluated retrospectively using information available from the records of nursing notes, medical prescriptions, and patient water balance, based on information from the last 24 hours.

Hemoglobin, serum urea, and creatinine levels; arterial lactate; the need for blood products; medications used; requiring mechanical ventilation for >48 hours, neurological or cardiovascular complications, the need for dialysis, and the need for performing a diagnostic test outside the ICU were registered. Routine laboratory tests (urea, serum creatinine, electrolytes, and liver profile) available in the patient’s medical records and those conducted in the ICU were obtained to record the outcomes. Therefore, there were no additional costs to the institution in conducting this study. After day 7 of hospitalization, if the patients remained in the hospital unit, the researchers continued monitoring them until they were considered fit for discharge, died, or were transferred to another institution; however, examination records were no longer recorded after day 7 of hospitalization.

All researchers involved in data collection received training from the principal investigator regarding the collection procedures. A 30-day pilot test was also conducted before data collection to ensure that necessary adjustments were made in the event of any divergences.

Primary outcomes were mortality, length of ICU stay, and length of hospital stay (LHS). Secondary outcomes were the need for dialysis, pressure injury, acute kidney injury (AKI), acute myocardial infarction, stroke, mechanical ventilation for >48 hours, infection, and hospital readmission.

Data were collected daily by data collection assistants, who were scheduled to visit the ICUs of both hospitals every day. This was to ensure that two assistants, at the minimum, were present at the ICUs every day of the week and that all information necessary for the study was collected from the patients’ medical records. After the assistants completed the instruments, which lasted for 4 hours, pilot data collection was started, which lasted for 1 month. Next, the data collection instruments were audited to verify the quality of information obtained and make necessary adjustments for possible failures in data acquisition.

The data obtained were plotted in tables using Microsoft Excel 2010. Categorical variables were presented as absolute and relative percentage frequencies. Continuous variables were presented as mean, median, standard deviation, and interquartile range. Fisher’s exact, Pearson’s χ^2^, and Pearson’s χ^2^ tests with Monte Carlo simulations were applied to assess the association between categorical variables. The Shapiro–Wilk test was used to assess the normal distribution of continuous variables. The Mann–Whitney U test was used to assess the differences in measures of central tendency. Linear regression was used to analyze continuous variables, and logistic regression was used for binary and multinomial variables in the confounding models. In addition, the coefficient of determination, adjusted coefficient of determination, area under the curve, sensitivity (rate of true positives), and specificity (rate of true negatives) were used to assess the goodness-of-fit of the proposed models.

In this study, workload score (NAS), prognosis (SOFA and SAPS 3), and outcomes (death, pressure injury, dialysis, infection, length of stay in the ICU [LSICU], LHS, and Kidney Disease: Improving Global Outcomes) were used because of their representativeness in understanding the hospitalization of patients in the ICU in different hospital environments. Regarding the application of the models, two forms were used: the general model (hospital), which contained all variables considered in this study as independent variables, and the conditioned model, which had a choice of variables (p<0.2 in the univariate analysis) that had a greater influence on the outcomes analyzed according to the knowledge of the researchers. We adopted a 5% level of significance. All analyses were performed using R statistical software (R Core Team 2020).

This study was approved by the Research Ethics Committee of the *Universidade Federal de Sergipe* (CAAE: 92517018.0.0000.5546; # 2,830,187).

## RESULTS

Overall, 219 patients were included in this study. A comparative analysis of the clinical and demographic characteristics between patients in H1 and H2 showed that both groups were similar, except that white race (47.4% *versus* 69.5%, p=0.001) was more predominant in H2, and patients in H1 had higher body weight (62.8±12.8kg *versus* 54.9±10.2kg, p<0.001) than those in H2. Furthermore, patients in H1 had more comorbidities than those in H2, including severity at admission, as assessed by the prognostic scores and the need for care for worse admission in the receiving hospital (Table 1).

**Table 1:** Clinical and demographic characteristics of patients

Variables	H1 (n=101)	H2 (n=118)	p value
Age in years, mean±SD	62.7±16.1	56.6±21.6	0.087^†^
Sex, n (%)			
Male	56.0 (55.4)	63.0 (53.4)	0.787^£^
Female	45.0 (44.6)	55.0 (46.6)	
Race, n (%)			
White	46.0 (47.4)	82.0 (69.5)	0.001^£^
Black	51.0 (52.6)	36.0 (30.5)	
Weight (Kg), mean±SD	62.8±12.8	54.9±10.2	<0.001^†^
Diagnosis by systems, n (%)			
Neurological	20.0 (20.0)	26.0 (22.0)	0.288^#^
Respiratory	38.0 (38.0)	45.0 (38.1)	
Cardiovascular	6.0 (6.0)	14.0 (11.9)	
Digestive	12.0 (12.0)	9.0 (7.6)	
Renal	0.0 (0.0)	3.0 (2.5)	
Endocrine-metabolic	4.0 (4.0)	9.0 (7.6)	
Hematological	1.0 (1.0)	1.0 (0.8)	
Neoplasm	2.0 (2.0)	1.0 (0.8)	
Infection	13.0 (13.0)	7.0 (5.9)	
Trauma	4.0 (4.0)	3.0 (2.5)	
Heart failure, n (%)	15.0 (16.7)	13.0 (11.0)	0.306^‡^
Previous AMI, n (%)	11.0 (12.0)	13.0 (11.0)	0.831^‡^
Systemic arterial hypertension, n (%)	51.0 (54.8)	37.0 (31.4)	0.001^£^
Dyslipidemia, n (%)	12.0 (13.0)	9.0 (7.7)	0.248^‡^
Current smoker, n (%)	22.0 (23.9)	8.0 (6.8)	0.001^‡^
Basal creatinine >1.5, n (%)	40.0 (43.5)	13.0 (11.0)	<0.001^‡^
Diabetes, n (%)	35.0 (37.2)	21.0 (17.9)	0.003^‡^
Previous CVA, n (%)	21.0 (22.8)	10.0 (8.5)	0.005^‡^
ICU admission support, n (%)			
Use of dobutamine	7.0 (7.2)	3.0 (2.5)	0.191^‡^
Use of noradrenaline	45.0 (45.5)	10.0 (8.5)	<0.001^‡^
Use of fentanyl	68.0 (67.3)	61.0 (51.7)	0.020^£^
Use of midazolam	49.0 (49.0)	25.0 (21.2)	<0.001^‡^
NGT, n (%)	55.0 (56.7)	74.0 (62.7)	0.403^£^
Orotracheal tube, n (%)	72.0 (74.2)	55.0 (47.0)	<0.001^£^
Central venous catheter, n (%)	48.0 (49.5)	43.0 (36.4)	0.054^£^
Drains, n (%)	18.0 (18.6)	7.0 (5.9)	0.005^‡^
IUC, n (%)	81.0 (84.4)	101.0 (85.6)	0.849^‡^
SOFA 24 hours, mean±SD	4.4±3.0	4.0±4.9	0.061^†^
SOFA daydis, mean±SD	4.5±4.2	2.9±3.8	0.002^†^
SAPS 3 D1, mean±SD	34.6±13.7	29.4±13.5	0.011^†^
SAPS 3 daydis, mean±SD	33.1±16.9	24.2±14.2	<0.001^†^
NAS 24 hours, mean±SD	49.3±11.7	44.0±8.9	0.002^†^
Charlson, mean±SD	3.5±2.1	2.4±2.2	<0.001^†^

^†^ Mann-Whitney test; ^‡^ Fisher’s exact test. ^£^ Pearson’s χ^2^ test. ^#^ Pearson’s χ^2^ test with Monte Carlo simulations.

SD: standard deviation; AMI: acute myocardial infarction; CVA: cerebrovascular accident; IUC: indwelling urinary catheter; NGT: nasogastric tube; SOFA: Sequential Organ Failure Assessment; D1: first day in the ICU; SAPS: Simplified Acute Physiology Score; NAS: Nursing Activities Score; ICU: intensive care unit.

A comparison of the ICU outcomes between patients admitted to H1 and H2 (Table 2) showed that the percentage of deaths (58.0% *versus* 24.1%, p<0.001), need for dialysis (23.0% *versus* 5.9%, p<0.001), development of pressure injuries (30.3% *versus* 2.6%, p<0.001), need for mechanical ventilation for >48 hours (71.7% *versus* 38.5%, p<0.001), and infection (58.8% *versus* 33.1%, p<0.001) were higher in H1 than in H2. In addition, the mean LHS was longer in patients admitted to H1 (26.0±19.0 days *versus* 14.8±16.6 days, p<0.001) than to H2.

**Table 2:** Clinical outcomes of patients

Outcomes	H1 (n=101)	H2 (n=118)	p value
Death, n (%)	58.0 (58.0)	28.0 (24.1)	<0.001^£^
Dialysis, n (%)	23.0 (23.0)	7.0 (5.9)	<0.001^‡^
Pressure injury, n (%)	30.0 (30.3)	3.0 (2.6)	<0.001^‡^
AKI, n (%)	44.0 (43.6)	27.0 (36.5)	0.346^£^
AMI	7.0 (7.1)	3.0 (2.5)	0.191^‡^
CVA, n (%)	10.0 (10.2)	7.0 (5.9)	0.312^‡^
Mechanical ventilation >48 h, n (%)	71.0 (71.7)	45.0 (38.5)	<0.001^£^
Infection, n (%)	57.0 (58.8)	39.0 (33.1)	<0.001^£^
LSICU, mean±SD	16.7±15.6	12.8±12.2	0.051^†^
LHS, mean±SD	26.0±19.0	14.8±16.6	<0.001^†^
Readmission to ICU, n (%)	1.0 (1.2)	5.0 (4.3)	0.404^‡^

^†^ Mann–Whitney test. ^‡^ Fisher’s exact test. ^£^ Pearson’s χ^2^ test.

SD: standard deviation; AKI: acute kidney injury; AMI: acute myocardial infarction; CVA: cerebrovascular accident; LSICU: length of stay in the intensive care unit; LHS: length of hospital stay; ICU: intensive care unit.

The logistic regression analysis performed to assess death outcome showed that patients in H1 were 3.14 times more likely to die than those in H2; however, when adjusted for age, SAPS 3, SOFA Score, origin, infection, ICU readmission, mechanical ventilation, LSICU, dialysis, AKI, and NAS, no significant statistical difference was observed. Patients admitted to the H1 ICU were 14 times more likely to develop pressure injuries than those admitted to H2. However, even after adjusting for age, sex, mechanical ventilation, NAS, and SAPS 3, lesion appearance remained 11.5 times greater in H1 than in H2 (Table 3).

**Table 3:** Logistic regression analysis performed for death and pressure injury of the evaluated patients

	H2	H1 OR (95% CI)	p value	AUC	SE	SP
Deaths (n=145)						
Hospital	1	3.14 (1.59–6.31)	<0.001	0.634	0.700	0.573
Hospital, adjusted for age, SAPS 3, SOFA, origin, infection, readmission to the ICU, mechanical ventilation, LOS-ICU, dialysis, AKI, and NAS	1	2.78 (0.94–8.28)	0.065	0.800	0.802	0.797
Pressure injury (n=191)						
Hospital	1	14.02 (4.61–61.03)	<0.001	0.869	0.869	0.000
Hospital, adjusted for age, sex, mechanical ventilation, NAS, and SAPS 3	1	11.55 (3.04–43.96)	<0.001	0.895	0.901	0.778

OR: odds ratio; 95%CI: 95% confidence interval; AUC: area under the curve; SE: sensitivity; SP: specificity; NAS: Nursing Activities Score; SAPS 3: Simplified Acute Physiology Score 3; SOFA: Sequential Organ Failure Assessment’ ICU: intensive care unit; AKI: acute kidney injury; LOS-ICU: length of stay in intensive care unit.

**Figure 1:**
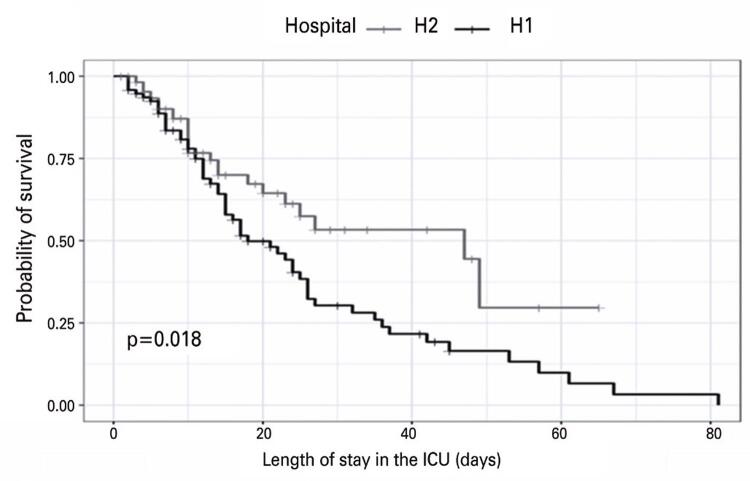
Survival curve of patients evaluated in the study hospitals as a function of the length of stay in the intensive care units estimated using the log-rank test

Figure 1 shows the survival curves for the ICU stay. For the first 15 days, no significant difference between both institutions was observed; however, from day 20 onwards, the curves changed significantly. The H2 curve indicated that the patients had a shorter length of stay in the ICU, with a higher survival rate and lower mortality.

ICU: intensive care unit.

**Figure 2:**
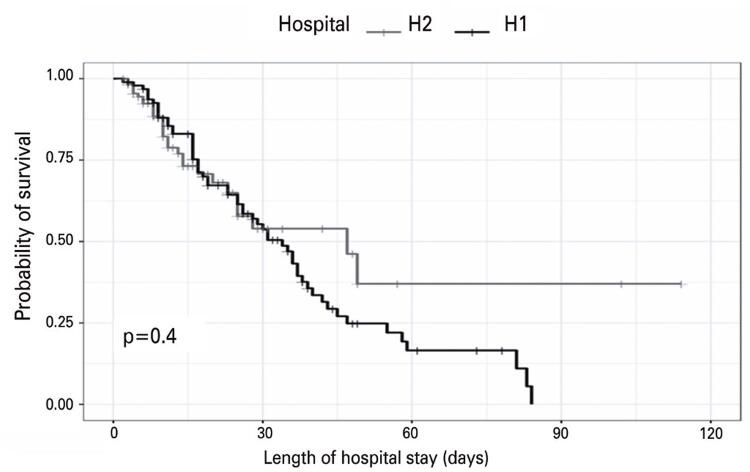
Survival curve of patients evaluated in the study hospitals as a function of the length of hospital stay estimated using the log-rank test

Length of hospital stay evaluation indicated that survival was similar in both institutions until day 30 of hospitalization. As shown in figure 2, the ICU survival rate in H1 decreased after day 60 and in H2 after approximately day 55 of hospitalization. However, only 13% of patients admitted to the H2 ICU were discharged from the hospital between day 30 and day 50, and after day 50, no deaths were observed.

## DISCUSSION

In this study, we found that patients admitted to H1 ICU, despite not showing a significant difference concerning admission diagnosis, were more prone to systemic arterial hypertension, had a higher prevalence of chronic kidney disease (admission creatinine >1.5mg/dL), diabetes, and previous stroke. At ICU admission, H1 patients needed more support, had worse prognostic indices (SAPS 3 and Charlson), and had a higher nursing workload in the first 24 hours of admission. In addition, worse outcomes, including mortality, need for dialysis, pressure injury, infection, longer mechanical ventilation time, and longer hospital stay, were observed in patients admitted to H1 than to H2.

Although some studies have reported improvements in severe sepsis-related outcomes in the ICU despite the lack of new therapies, the reduction in mortality may be attributed to advances in care processes,^(14-16)^ such as early antibiotic administration^(17,18)^ shock resuscitation strategies,^(19)^ protective mechanical ventilation,^(20)^ and increased availability of specialists in the intensive care.^(21)^ It is believed that ICUs linked to teaching hospitals with a high volume of critically ill patients may have more effective care processes, resulting in lower rates of hospital mortality than centers with a low volume and without academic links.^(22)^ However, this finding was not confirmed at the time of conducting this study, considering the patients included in H1, and the reasons for the worst outcomes will be discussed below.

Compared with other studies,^(23-26)^ which also evaluated patients admitted to ICUs outside urban centers, the participants admitted to the ICUs in this study had higher mortality rates and worse outcomes. This may be because hospitals located outside large centers usually have a lower volume of cases than urban hospitals. Low ICU case volume has been associated with poor outcomes globally, particularly in patients with moderate disease severity and those requiring mechanical ventilation during hospitalization.^(1,22,27-29)^

In ICUs with high hospitalization volumes, better outcomes have been reported, particularly in specialized units, such as coronary care units^(30)^ and respiratory ICUs.^(31)^ This association between high volumes of hospitalization and favorable outcomes may be attributed to the characteristics of the receiving units, such as access to more resources, more experienced teams, a higher nurse-bed ratio,^(31)^ and the use of evidence-based protocols.^(32)^ In H1 ICU, where the worst outcomes were observed, in addition to the absence of the above-mentioned characteristics, the focus was on the fact that the care teams were composed a short time ago and there were no institutional care protocols.

However, the data from the present investigation contrast the results previously presented by our group, which was a cross-sectional study including two ICUs of another teaching hospital located in the capital of the same state, with a mortality rate of 21%.^(33)^ In these ICUs where the volume of admissions was high, the teams comprised experienced professionals and used updated care protocols, justifying the best results concerning this investigation.

The SAPS 3 and Charlson Scores assessed at patient admission were higher in H1 than in H2, reflecting greater admission severity. This finding, in addition to the absence of updated care protocols and trained staff at H1, as also observed by other authors,^(14-16)^ may explain the higher prevalence of unfavorable outcomes in H1. In a retrospective cohort study conducted to characterize the level of exacerbation, admission severity, and intensity of care and to identify the predictors of severity, the authors observed an association between high disease burden (assessed using CCI) and worse prognosis (measured by SAPS 2 and Logistic Organ Dysfunction System) at hospital admission with a high probability of worse outcomes.^(34)^

The findings of our study also showed that patients admitted to H1 ICU had higher SAPS 3 values than those admitted to H2 ICU. This may be attributed to the characteristics of the patients admitted to H1, including age >60 years, hypertension (mostly from the emergency unit), increased use of vasoactive drugs, and increased use of invasive devices. In a retrospective cohort study, Jahn et al. showed that patients with higher SAPS 2 and 3 values at admission were more prone to death.^(35)^

In addition to greater severity, H1 patients had a higher workload. The association between higher admission severity and higher nursing workload has been previously reported.^(36)^ Romano et al. showed that SAPS 3 values were predictors of a higher nursing workload.^(37)^ Similarly, Oliveira et al. also showed that the NAS of patients admitted to an ICU located in the city of São Paulo was associated with Charlson Score, SAPS 3, LHS, and LSICU.^(36)^As NAS was calculated retrospectively based on the nursing records in the last 24 hours, the greater workload presented by these patients may be due to the greater need for attention required to achieve stability of organic functions.

Patients admitted to the ICU are susceptible to complications such as pressure injuries. In this study, a higher incidence of pressure injury was observed in H1, which was 14 times greater than in H2, and remained high even when adjusting for other variables. This may have been influenced by factors such as comorbidities, SAPS 3 Scores, and high nursing workloads, as assessed using NAS. A study suggested that age, use of noradrenaline, and mechanical ventilation for more than 72 hours are factors associated with a higher incidence of pressure injury, as observed in this investigation.^(38)^ Such factors, when combined in an intensive care environment, create an atmosphere conducive to the emergence of lesions, because noradrenaline acts as a vasoconstrictor, which reduces blood supply to the periphery. Prolonged mechanical ventilation limits the patient’s movement in bed due to sedation, reducing sensory perception. In addition, a reduction in skin thickness and a decrease in dermal capillaries are observed in elderly patients. It is noteworthy to mention that the high nursing workload, resulting in the lack of time to frequently change the patient’s position, contributes to the emergence and development of injuries.

Based on the existing literature, the most current evidence comes from nonrandomized studies. Furthermore, patient populations in different types of hospitals can be heterogeneous; for example, teaching hospitals usually receive the most complex cases.^(2)^ The physical structure and availability of technology may differ between teaching and nonteaching hospitals.^(39,40)^ In addition, health structure may differ in terms of the characteristics of processes, such as measures that address the proper implementation of healthcare. Although these are not clinical outcomes evaluated in patients, they can be translated into differential outcomes; precisely, if the most appropriate treatment is used more frequently, patient outcomes tend to be better.^(41)^

Nonetheless, this study has some limitations. The transition of the clinical governance model observed in H1, which made it difficult to implement and use protocols guided by evidence-based practices, combined with the lack of experience of the ICU teams, may have influenced the worst results.

The findings of our study suggested that the need for teaching hospitals to present well-established care protocols is critical to impart relevant, accurate, and current information to students and guarantee the evidence-based quality of care to patients.

## CONCLUSION

Worse outcomes, including death, dialysis, pressure injury, acute kidney injury, need for mechanical ventilation for >48 hours, infection, and length of hospital stay, were more prevalent in the teaching hospital than in the hospital without academic affiliation. There was no difference between the institutions concerning the survival rate of patients as a function of the general length of hospital stay; however, this difference was observed concerning intensive care units admissions.
